# Wheat and Flour

**Published:** 1876-10

**Authors:** M. L. Holbrook

**Affiliations:** Editor of Herald of Health, Author of “Eating for Strength”


### *WHEAT AND FLOUR.*

BY DR. M. L. HOLBROOK,

Editor of Herald of Health, Author of "Eating for Strength."

Wheat is one of the most important articles of food, and probably used more extensively than any other grain. A kernel of wheat, as it comes out of the chaff or husk, is an interesting subject for thought. In a rough analysis it is composed of two parts, the colored, tough, tegumentary skin, and the easily pulverized interior, which we call flour. This tegumentary exterior layer is of so dense and woody a nature, and so tough, that it forms into a scale when ground. It is perfectly indigestible, and serves no purpose as a nutriment. Its effect on the bowels is to irritate the alimentary canal, hurry the food through quickly, and with it valuable nutriment escapes without undergoing digestion. Beneath this external skin lies a soft, friable cortex, which in the ordinary process of grinding escapes with the tough, tegumentary portion. This part is very valuable nourishing material, as it is rich in nitrogenous matter, fat, and salts. Bran, then, consists of two parts, the tough, worthless part, and that next to it, the very best part of the wheat. Still deeper in the kernel is another part, which is white, and composed mostly of starch. White flour, as ordinarily eaten, is composed of this inner white, and largely starchy substance.

Graham flour contains all three parts, and is altogether to be preferred to the white, if made of good white wheat, and free from the tough cuticle. A new method has been discovered of preparing flour from which this external cuticle has been removed, but not with it the gluten layer just below it, which is really the most valuable part of the wheat. Flour made in this way is vastly better than white flour, for the latter is too starchy, and not sufficiently rich in nitrogenous, fatty, and mineral matter. It is also much to be preferred to flour made with the whole of the external cuticle retained, for this is not nourishing, nor in any way useful, and may be is often injurious. We hail all improvements in flour as real benefactions to our race. We hailed the advent of whole wheat flour, which, though objectionable on account of the tough, tegumentary portion, is rich in gluten, fat, and mineral matter. We hail now again the new improvement, because it makes the PERFECT FLOUR, free, as it is, from the excess of starch and deficiency of mineral matter, and free also from the injurious woody skin of the wheat.

It is this latter flour which the Health Food Co. are so earnest in introducing into use. They deserve the thanks of their fellow-men, and above all the thanks of that large class of sedentary brain-toilers who need in their food those elements which hold the nervous system in the best condition for good work. Long may they prosper.

